# Low-Dose Aspirin Prevents Kidney Damage in LPS-Induced Preeclampsia by Inhibiting the WNT5A and NF-κB Signaling Pathways

**DOI:** 10.3389/fendo.2021.639592

**Published:** 2021-03-11

**Authors:** Guanlin Li, Wei Wei, Lingge Suo, Chun Zhang, Haiyan Yu, Hui Liu, Qing Guo, Xiumei Zhen, Yang Yu

**Affiliations:** ^1^ Clinical Stem Cell Research Center, Peking University Third Hospital, Beijing, China; ^2^ Department of Ophthalmology, Beijing Key Laboratory of Restoration of Damaged Ocular Nerve, Peking University Third Hospital, Beijing, China; ^3^ Center for Reproductive Medicine, Department of Obstetrics and Gynecology, Peking University Third Hospital, Beijing, China

**Keywords:** aspirin, lipopolysaccharide, kidney injury, WNT5A/NF-κB, preeclampsia

## Abstract

Preeclampsia (PE) is a serious pregnancy-related disease, and patients usually present with a high inflammatory response. Previous studies have suggested that aspirin (ASP) may have a role in alleviating the pathogenesis of preeclampsia. However, whether ASP can improve kidney damage and the mechanism for improving it is currently unclear. Here we optimized a lipopolysaccharide (LPS)-induced PE mouse model to identify the role of ASP in renal protection. We found that ASP treatment ameliorated LPS-induced renal failure and pathological changes, the tubular injury was significantly attenuated by ASP. Administration of ASP decreased the renal expression of pro-inflammatory factors, resulting in reduced kidney inflammation. The number of GALECTIN-3-positive cells was reduced, and the up-regulation of IL-6 and TNF-α was decreased. In addition, ASP also suppressed renal cell apoptosis and oxidative stress. An *in vitro* study indicated that ASP relieved LPS-induced HK-2 cell damage by inhibiting WNT5A/NF-κB signaling. Collectively, our data suggest that ASP is a useful therapeutic option for PE-related kidney injury.

## Introduction

Preeclampsia (PE) is a serious pregnancy-related disorder and a leading cause of fetal and maternal morbidity and mortality (1, 2). This multisystem disorder is characterized by new-onset hypertension, proteinuria, and inflammation, and generally appears after the 20th week of gestation (3, 4). Although the pathogenesis is uncertain, the disease is widely hypothesized to be related to an abnormal maternal inflammatory response (5).

Inflammatory cytokines are major influencing factors of the kidneys during pathological pregnancy, which mainly lead to swollen glomeruli, fibrin deposits, and capillary occlusion (6, 7). Reducing the production of pro-inflammatory factors and suppressing downstream signaling may constitute an effective approach to treat pregnancy-related kidney injury. However, how inflammatory signaling can be regulated in PE-related kidney damage is currently unknown.

Aspirin (ASP) is an anti-inflammatory drug that is widely utilized for clinical treatment (8, 9). Research shows that ASP given in low doses (60–150 mg/day) is an effective prevention for preeclampsia and fetal growth restriction (10–13). Early treatment with low-dose ASP has been shown to reduce the incidence of early-onset PE in high-risk women (10, 14, 15). Our previous study demonstrated that low-dose ASP ameliorated the PE-like phenotype in a lipopolysaccharide (LPS)-induced mouse model by inhibiting the NF-κB pathway, thus exerting an anti-inflammatory effect (16).

The WNT signaling pathway is classified as both a canonical and non-canonical pathway (17), with critical roles in complex cellular responses through a highly organized network involving ligands, receptors, and downstream mediators (18, 19). The signaling pathway serves as a dominant regulator in various diseases (20–22). Increasing evidence has demonstrated a relationship between the WNT signaling pathway and inflammation (23, 24). WNT signaling triggers both anti-inflammatory and pro-inflammatory functions partially by either repressing or enhancing the NF-κB pathway, respectively (25). Previous study identified the regulation of LPS in the classical WNT/β-catenin signaling in HK-2 cells (26), and the function of WNT/β-catenin signaling in LPS-induced podocyte injury also reported (27). However, the effects of non-classical WNT signaling were rarely reported. WNT5A, a typical glycoprotein of the non-canonical pathway, has been verified to have pro-inflammatory functions in urosepsis, nephrogenic diabetes insipidus (NDI), and chronic kidney disease (CKD) (28–30). WNT5A signaling play potential roles in human placental and malfunction of it can cause preeclampsia (31, 32). So the critical function of WNT5A in preeclampsia related kidney injury is worth studying.

Although the abnormalities in PE kidneys, such as swollen glomeruli, fibrin deposits, and capillary occlusion, have been well clarified, the regulatory mechanisms are still poorly understood. Therefore, we speculated that low-dose ASP may regulate PE-related kidney injury *via* the WNT5A and NF-κB signaling pathways. To investigate these assumptions, we improved a pre-established PE-like mouse model to study the regulation of ASP on renal injury. The PE-related phenotype, renal cell apoptosis, and oxidative stress in LPS-treated mice were examined, and the roles of ASP in the kidney were evaluated. An *in vitro* study confirmed that ASP relieved LPS-induced HK-2 cell damage by inhibiting WNT5A/NF-κB signaling. We disclose a novel regulatory mechanism of ASP intervention in PE-related kidney injury. Our findings provide evidence that ASP is a useful drug for preventing kidney injury associated with PE.

## Materials and Methods

### Cell Culture and Treatment

The HK-2 human kidney tubular epithelial cell line was attained from the American Type Culture Collection (ATCC) and cultured in Dulbecco’s Modified Eagle’s Medium/Nutrient Mixture F-12 (Gibco, USA) containing 10% fetal bovine serum at 37°C in a cell incubator with 5% CO_2_. Before treatment with LPS, ASP (Sigma Aldrich, USA) and the WNT5A antagonist Box-5 (Merk Millipore, USA), cells were starved for 24 h in medium containing 0.5% FBS. For the treatment experiments, cells were treated with LPS (10 μg/ml) alone for 24 h or ASP (5 × 10^−4^ M) 12 h after LPS treatment. Box-5 (200 μM) was added at the same time as ASP.

### Animals and Experimental Protocols

All animal studies were reviewed and approved by the Animal Ethics Committee of Peking University Health Science Center. Systemic LPS was used to induce a PE-like pregnant mouse model, and low-dose ASP was administered, as previously described (16). Given that aspirin is most effective before 16 weeks of pregnancy in humans (33), we further optimized the animal model for ASP treatment at embryonic day 9.5 (E9.5) (for the previous study, ASP treatment was at E7.5), probably corresponding to gestational week 13 in humans (34, 35). Briefly, CD1 Institute of Cancer Research mice (8 weeks old) were used in our study. Experiments were performed in the following groups of mice: control group (CTRL, N = 20), LPS-treated group (N = 20), ASP-treated group (N = 20), and LPS + ASP co-treatment group (N = 20). Mice in the CTRL group were treated with saline according to the volume of LPS or ASP. For the LPS-treated group, 20 μg/kg LPS (Sigma Aldrich, USA) was intraperitoneally injected daily from E7.5 to E17.5. ASP (Sigma Aldrich) at a dose of 15.2 mg/kg dissolved in saline was intragastrically infused 2 h after LPS administration daily from E9.5 to E17.5. According to the harmacological experimental methodology, the equivalent dose ratio of mice to humans was 9.1 (doses are expressed in milligrams per kilogram). ASP intervention in mice with a dose equivalent to 100 mg/day in pregnant women with an average weight of 70 kg. All mice were sacrificed at E18.5 prior to delivery, and their serum, urine, andorgans (placentas and kidneys) were collected.

### Blood Pressure and Urinary Protein Levels

The systolic blood pressure (SBP), mean blood pressure, and diastolic blood pressure (8:00 a.m.–11:00 a.m.) of each mouse were monitored daily using a tail-cuff plethysmograph (Softron, Japan). All mice were acclimatized to blood pressure procedures 5 days prior to pregnancy. Urine from each mouse was collected on E18.5 with dams housed individually in metabolic cages with no food. The urinary albumin levels were measured by an Albumin Assay Kit (Bethyl Laboratories, USA), and the urinary creatinine concentrations were determined using a Creatinine Assay Kit (Cayman Chemical, USA). The ratio of urinary albumin to creatinine was calculated as the proteinuria index, as previously described (36).

### Blood Collection

Mice were fed in a restrainer where the temperature was maintained at 24°C to 27°C. They were placed in an appropriate restraint device with their tails extended, and a small nick over the lateral tail vein was made using a sterile scalpel blade. A collection tube was used to collect blood. Once a sufficient amount of blood was collected, the nick was pressed with clean gauze to stop the flow of blood. For the LPS-treated group, peripheral blood was collected 4 h after LPS treatment. For the LPS +ASP and ASP groups, ASP was administered 2 h after LPS treatment. Blood was collected 2 h after ASP administration at E10.5, E13.5 and E18.5.

### Isolation and Culture of Kidney Primary Cells

The kidney tissue was minced with scalpel, and the fragments were incubated at 37°C in collagenase-trypsin solution. Cells were washed and centrifuged and isolated kidney cells were cultured in Dulbecco’s Modified Eagle’s Medium/Nutrient Mixture F-12 (Gibco, USA) containing 10% fetal bovine serum at 37°C in a cell incubator with 5% CO_2_. The cells were obtained from pregnant mice at E18.5 in CTRL, LPS, L+A and ASP treatment group, and six animals from each group respectively.

### Hematoxylin and Eosin (H&E), Periodic Acid-Schiff (PAS) Staining and Immunohistochemical Staining in Placental and Kidney Tissues

sPlacental and kidney tissues were harvested from the mice on E18.5, fixed in 4% paraformaldehyde overnight at 4°C and embedded in paraffin wax. Paraffin sections at a 5-μm thickness were routinely dewaxed and rehydrated, stained with H&E using the standard techniques and analyzed. Histological quantification of the number of calcifications per field was performed under 10× magnification in a random field (18 placentas for each group). The statistical analysis was based on the results of six animals from each group and three placentas from each animal.

For PAS staining, renal injury was assessed by quantifying the glomeruli that showed characteristic features of damage, which mainly included a decreased Bowman’s space and occlusion of the capillary loop spaces. Histological quantification of renal damage per field was performed under 10× magnification in a random field (15 glomeruli for each field and 6 animals/kidneys for each group). The glomeruli in each field were given a score based on the amount of capillary space evident within Bowman’s capsule, as described previously (16). The highest score of 5 was awarded to a glomeruli patient with a normal amount of interstitial space within Bowman’s capsule. A score of 1 was assigned to glomeruli that showed a complete loss of capillary space, and an intermediate score of 3 was assigned to glomeruli that displayed reduced, but not completely erased, capillary spaces. The scores for each field were divided by the number of glomeruli to obtain an average score per glomerulus for each field.

For immunohistochemical staining, sections were subjected to antigen retrieval before being incubated with antibodies against mouse NGAL, GALECTIN-3 (Abcam, USA), and KIM-1 (Cell Signaling Technology, USA). A negative control was set by replacing the primary antibody with preimmune IgG. The sections were further incubated with an appropriate secondary antibody conjugated to horseradish peroxidase and visualized with diaminobenzidine (Zhongshan Golden-bridge Biotechnology, China) as the substrate. All slides were scanned using an Olympus IX71 microscope equipped with a DP72 digital camera system and analyzed with Image-Pro Plus version 6 software.

### Terminal Deoxynucleotidyl Transferase-Mediated Deoxyuridine Triphosphate Nick-End Labeling (TUNEL) Staining

TUNEL assay (Roche Diagnostics, USA) was used to measure cell apoptosis in the kidneys according to the manufacturer’s instructions. Briefly, the sections were dewaxed, rehydrated, reacted with proteinase K, and washed with PBS. Samples were then incubated in the TUNEL reaction mixture. For the positive control, samples were treated with RNase-free DNase I (Invitrogen, USA) and incubated with the TUNEL reagent. All TUNEL-positive cells on a whole section were counted in a blinded manner in 10 randomly chosen fields (20× magnification) per kidney.

### Oxidative Stress Markers and Antioxidant Assays

The level of Reactive oxygen species (ROS) was measured in the homogenate samples using 2’, 7’-dichlorodihydrofluorescein diacetate (H_2_DCF-DA) (Sigma Aldrich, USA). The fluorescence intensity was measured at an excitation wavelength of 490 nm and an emission wavelength of 540 nm using a plate reader. Malondialdehyde (MDA), reduced glutathione (GSH), superoxide dismutase (SOD), and catalase (CAT) were evaluated by corresponding detection kits according to the manufacturer’s protocol (Nanjing Jiancheng Bioengineering Institute, China).

### ELISAs

Mice plasma concentrations of IL-6, TNF-α, and sFLT-1 were measured using the Mouse TNF-α Quantikine ELISA Kit, the Mouse IL-6 Quantikine ELISA Kit, and the Mouse VEGF R1/FLT-1 Quantikine ELISA Kit (all from R&D Systems, Australia), according to the manufacturer’s instructions. The concentrations were determined according to the absorbances of the samples and standards at a wavelength of 450nm obtained using a microplate reader (BioTek, USA).

### RNA Preparation and Quantitative Real-Time PCR

Total RNA was extracted with TRIzol reagent (Invitrogen, USA) and reverse transcribed into cDNA using the oligo deoxy thymidine primer (Amersham Biosciences, USA). Real-time PCR was carried out using the LightCycler480 sequence detection system (Roche, CH). The primer sequences were as follows: mouse TNF-α, 5-AGGCACTCCCCCAAAAGATG-3 (forward) and 5-TGAGGGTCTGGGCCATAGAA-3 (reverse); mouse IL-6, 5-CTCATTCTGCTCTGGAGCCC-3(forward) and 5-GACAGGTCTGTTGGGAGTGG-3 (reverse); mouse GAPDH, 5-CTCTTCCACCTTCGATGCCG-3 (forward) and 5- TTATGGGGGTCTGGGATGGA-3 (reverse), human TNF-α, 5- CACCACTTCGAAACCTGGGA-3 (forward) and 5- AGGAAGGCCTAAGGTCCACT-3 (reverse), human IL-6, 5- CTCAATATTAGAGTCTCAACCCCCA-3 (forward) and 5- ;GTGGGGCGGCTACATCTTT-3 (reverse), human GAPDH, 5-2 GAAGGTGAAGGTCGGAGTC-3 (forward) and 5- GAAGATGGTGATGGGATTTC-3 (reverse). All PCRs were performed in triplicate, and statistical analysis of the results was performed using the Ct value (Ct target gene–Ct GAPDH). The CT method of relative quantification was used to determine expression fold changes (37).

### Western Blotting Analysis

Total protein was extracted using radioimmunoprecipitation assay (RIPA) lysis buffer, and 40 μg of protein was subjected to 10% sodium dodecyl sulfate-polyacrylamide gel electrophoresis (SDS-PAGE) and subsequently electrotransferred to a nitrocellulose membrane (Amersham Pharmacia Biotech, UK). The membranes were incubated with antibodies against NGAL (Abcam, USA), KIM-1, cleaved CASPASE-3, BAX, BCL-2, cleaved PARP-1, p-NF-κB and NF-κB (all from Cell Signaling Technology, USA) as well as GAPDH (Ambion, USA) and then with a horseradish peroxidase-conjugated secondary antibody (Promega, USA). Visualization of the bands was achieved using the enhanced chemiluminescence western blot analysis system (Pierce, USA). The relative densities of the detected proteins were normalized to that of NF-κB or GAPDH in the same blot.

### Flow Cytometry

The apoptosis analyses by flow cytometer used Annexin V-FITC/PI double staining method (Solarbio, China), HK-2 cells were trypsin digested and collected with cold binding buffer (1 × 10^6^ cells/ml). Then, they added 5 μl Annexin V-FITC dyeing for 10 min, and 5 μl propidium iodide dyeing for 5 min in the dark. Samples were tested by using the Beckman CytoFLEX S flow cytometer (Beckman, USA).

### Statistical Analysis

The data are presented as the means ± standard errors of the mean (SEM). All the data were subjected to statistical analysis using 2-way analysis of variance (ANOVA) (for normally distributed data) and pairwise test with Bonferroni correction to determine the significance between groups. Differences were considered significant at P < 0.05. Statistical analysis was performed using GraphPad Prism 5 software (GraphPad Software, USA).

## Results

### ASP Diminishes Hypertension, Proteinuria, the Inflammatory Response, and Placental Dysplasia in Mice With LPS-Induced PE

**Figure 1:**
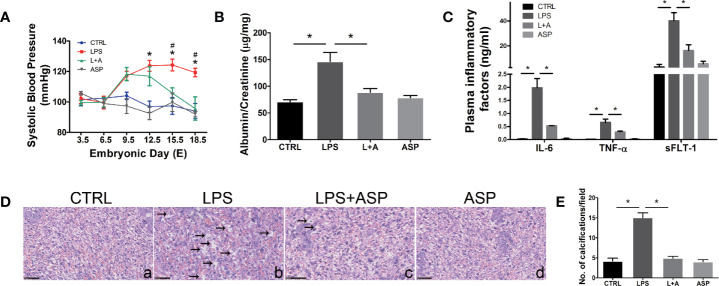
ASP rescued hypertension, proteinuria, inflammation, and placental dysplasia in mice with LPS-induced PE. **(A)** SBP was measured at daily intervals in each group. ^∗^LPS vs. CTRL. ^#^LPS vs. LPS + ASP; P < 0.05. **(B)** Ratio of urinary albumin to creatinine on E18.5 in each group. **(C)** Plasma IL-6, TNF-α, and sFlt-1 levels were detected by ELISA. The levels of TNF-α and IL-6 were measured on E10.5, and the level of sFLT-1 was measured on E18.5. **(D)** Placental H&E staining indicated that LPS-treated pregnant mice had damaged placentas; the black arrows show calcifications. ASP significantly attenuated placental damage. Scale bar, 25 μm. **(E)** Histological quantification of the number of calcifications. Data are expressed as the mean ± SEM. ^∗^Compared with the corresponding control. P < 0.05.

To study the function of ASP in PE-related kidney injury, a PE-like mouse model was established. Hypertension is the typical characteristic of PE. To determine whether LPS can contribute to gestational hypertension, we analyzed blood pressure in each group. The results indicated a significant increase in blood pressure in the LPS-treated group, and this increase was evident after 5 days (E12.5) of LPS injection. We found that LPS-induced hypertension was almost completely blocked by ASP (decrease from 121 to 97 mm Hg; P < 0.05 at E18.5). No significant blood pressure change was found in pregnant mice treated with ASP alone (**Figure 1A**). LPS-induced hypertension was prevented by co-treatment with ASP, suggesting that the LPS-induced increase in blood pressure was related to inflammatory pathway activation. In addition, LPS had no effect on blood pressure in non-pregnant mice, with or without ASP treatment (**Figure S1**).

Next, we evaluated the effect of ASP on proteinuria, another important feature of PE. Urine was collected on E18.5, and the albumin/creatinine level was measured by ELISA. The results indicated that ASP significantly reduced albumin/creatinine from 138 to 92 (P < 0.05). There was no significant change in the albumin/creatinine level in pregnant mice treated with ASP alone (**Figure 1B**). Overall, these findings provide *in vivo* evidence for the intervention effect of ASP on hypertension and proteinuria, the critical clinical features of PE. Furthermore, the levels of inflammatory factors in each group were analyzed. It was reported that the circulatory levels of IL-6, TNF-α, and sFLT-1 are elevated in PE patients (38). Our results indicated that LPS significantly increased IL-6, TNF-α, and sFLT-1 secretion in the peripheral blood of pregnant mice. ASP significantly inhibited LPS induction of these factors (**Figure 1C**), indicating the inhibitory effects of ASP on the inflammatory factors induced by LPS.

Placental H&E staining showed that LPS induced tissue damage, including significantly increasing the amount of calcification compared with that in the CTRL group (**Figure 1D**, a and b). ASP markedly reduced the calcification induced by LPS (**Figure 1D**, c). The placental structure of mice treated with ASP alone did not display any morphological changes (**Figure 1D**, d). Statistical results showed that the LPS-induced group had more calcified areas than the CTRL group, and that ASP could rescue this abnormal morphology (**Figure 1E**).

### ASP Attenuates Kidney Injury in Mice With LPS-Induced PE

To evaluate the pathogenic role of LPS in kidney damage and the effect of ASP, we further tested the morphogenesis of the tissue.

**Figure 2:**
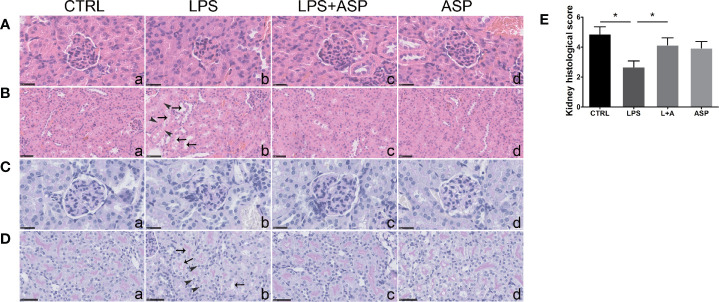
Effects of ASP on renal histology in mice treated with LPS. **(A, B)** H&E staining indicated that LPS induced kidney damage. The black arrows show tubular epithelial vacuolation and lumen dilation. The arrow heads show loss of epithelial cells. Scale bars, 25 μm in **(A)** and 50 μm in **(B)**. **(C, D)** Kidney PAS staining indicated that LPS-treated pregnant mice had damaged kidneys with typical endotheliosis with decreased capillary lumens with swollen endothelial cells. The black arrows indicate vacuolar deformation. The arrow heads show swelling of renal tubular epithelial cells. ASP significantly attenuated kidney injury. Scale bars, 25 μm in **(C)** and 50 μm in **(D)**. **(E)** The statistical results reflect arbitrary histological quantification of renal damage among the four groups. Data are expressed as the mean ± SEM. ^∗^Compared with the corresponding control. P < 0.05.

Kidney H&E staining indicated that the glomeruli of LPS-treated mice were smaller than those of the CTRL group, in which the glomeruli were open and easily distinguished (**Figure 2A**, a and b). In comparison, renal impairment was significantly reduced, despite not being completely eliminated, in the LPS+ASP group (**Figure 2A**, c). Moreover, tubular epithelial vacuolation, loss of epithelial cells, and dilation of the tubular lumen were observed in the LPS group (**Figure 2B**, a and b). ASP significantly attenuated renal tubular structural abnormalities (**Figure 2B**, c). The glomeruli and tubules of mice treated with ASP alone did not display any renal morphological changes (**Figures 2A, B**, d).

PAS staining of the kidney sections revealed that compared with the CTRL, LPS treatment induced histopathological alterations in the glomeruli, such as swollen glomeruli with narrowed capillaries and Bowman’s spaces (**Figure 2C**, a and b). Meanwhile, vacuolar deformation, swelling of tubular epithelial cells, and tubular lumen stenosis mainly occurred in the LPS group (**Figure 2D**, a and b). These changes can be rescued by co-treatment with ASP, while ASP alone had no effect on kidney damage (**Figures 2C, D**, c and d).

The statistical results showed the tubular injury scores in **Figure 2E**, according to the amount of capillary space within Bowman’s capsule. The scores of the LPS-treated group were much lower than those of the CTRL group. In the LPS +ASP group, higher scores were observed, as the reduced glomeruli and narrowed capillaries and Bowman’s spaces were attenuated. LPS did not cause kidney damage in non-pregnant mice (**Figure S2**).

**Figure 3:**
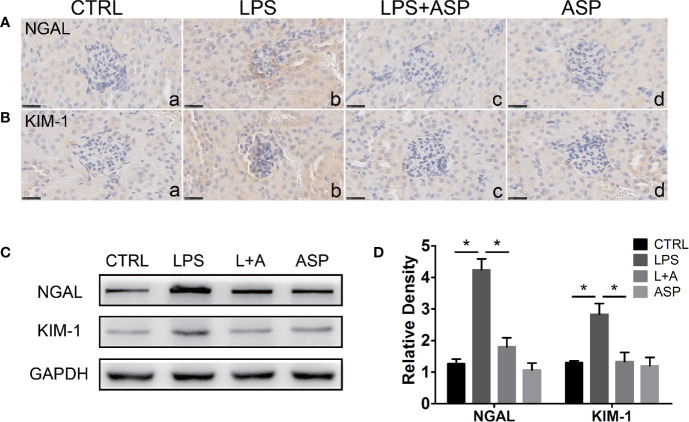
Effects of ASP on the renal expression of NGAL and KIM-1 in mice treated with LPS. **(A, B)** Immunohistochemical staining for NGAL and KIM-1 in kidney tissues. Scale bar: 25 µm. **(C)** Representative western blots showing the protein levels of NGAL and KIM-1 in the kidneys. **(D)** Bar charts showing the relative densities of the NGAL and KIM-1 bands. Data are expressed as the mean ± SEM. ^∗^Compared with the corresponding control; P < 0.05.

To further evaluate the rescue effect of ASP in tubule injury, we detected the expression of NGAL (neutrophil gelatinase-associated lipocalin) and KIM-1 (kidney-injury molecule-1) in the kidneys of each group. The two molecules are important markers of tubular injury and chronic kidney disease (39–41). Immunohistochemical staining revealed that administration of ASP significantly reduced the up-regulation of NGAL in the damaged tubules of mice treated with LPS (**Figure 3A**, a, b and c). Consistently, LPS-induced increases in the protein level of KIM-1 were markedly decreased by ASP (**Figure 3B**, a, b and c). The staining of NGAL and KIM-1 upon treatment with only ASP was not altered (**Figures 3A, B**, d).

Western blotting analysis showed the expression of NGAL and KIM-1, as the expression was significantly increased by 3.3-fold and 2.8-fold, respectively, in the LPS-treated group compared to the CTRL group. In the presence of ASP (L+A vs. LPS), the NGAL and KIM-1 levels were reduced by 51% and 46%, respectively. ASP treatment alone did not affect the expression of NGAL and KIM-1. These results indicate that ASP can attenuate kidney injury in mice with LPS-induced PE (**Figures 3C, D**).

### ASP Mitigates Oxidative Stress in LPS-Induced PE

**Figure 4:**
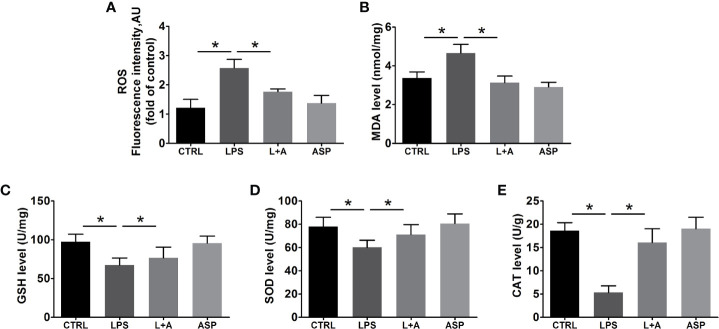
Effects of ASP on oxidative stress in the kidneys of mice treated with LPS. ASP attenuated oxidative stress injury according to biochemical parameters. ASP significantly reduced **(A)** ROS and **(B)** MDA contents and increased **(C)** GSH, **(D)** SOD, and **(E)** CAT contents in kidney tissues. Data are expressed as the mean ± SEM. ^∗^Compared with the corresponding control; P < 0.05.

Next, we evaluated the effect of ASP on the redox balance in the kidney in LPS-treated mice by determining ROS, MDA, and antioxidant defenses. As shown in **Figure 4A**, administration of LPS stimulated a significant increase (1.9-fold) in the production of ROS in the kidneys of mice compared with the CTRL condition. Consequently, the levels of MDA were significantly increased in LPS-treated mice (**Figure 4B**) by 1.3-fold. Co-treatment of the mice with ASP markedly prevented LPS-induced excess production of ROS and MDA, as the levels were reduced by 32% and 34%, respectively. Moreover, our data indicated that LPS can significantly damage the anti-oxidative capacity of kidney tissues. The levels of GSH, SOD, and CAT were decreased by 33%, 28%, and 61%, respectively. ASP administration effectively restored the levels of antioxidant markers in kidney tissue homogenates, with GSH elevated by 1.1-fold, SOD by 1.2-fold, and CAT by 3.4-fold (**Figures 4C–E**).

### ASP Inhibits Renal Tubule Apoptosis in LPS-Induced PE

**Figure 5:**
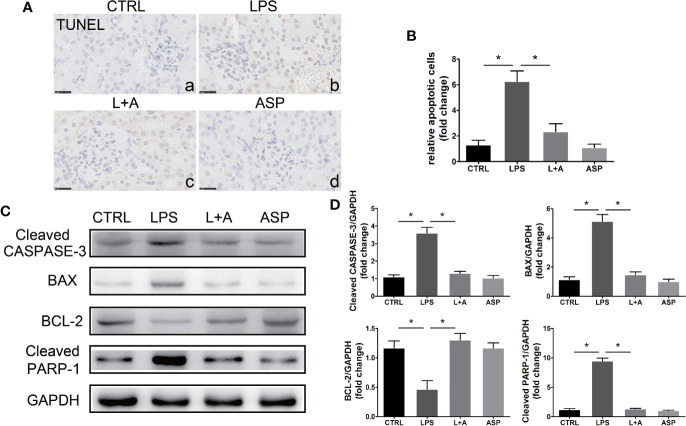
Effects of ASP on cell apoptosis in the kidneys of mice treated with LPS. **(A)** Representative images of TUNEL staining. Scale bar, 25 μm. LPS-treated pregnant mice had increased apoptosis in the kidneys, and ASP significantly reduced the number of apoptotic cells. **(B)** Histological quantification of the relative apoptotic cell number. **(C)** Representative western blots showing the protein levels of cleaved CASPASE-3, BAX, BCL-2, and cleaved PARP-1 in the kidneys. **(D)** Bar charts showing the relative densities of the cleaved CASPASE-3, BAX, BCL-2, and cleaved PARP-1 bands. Data are expressed as the mean ± SEM. ^∗^Compared with the corresponding control; P < 0.05.

To explore the effect of ASP on protecting the kidneys against apoptosis in a PE-like mouse model, kidney sections were stained with a TUNEL kit as described in the methods section. Compared to the CTRL treatment, LPS treatment induced extensive apoptosis in the kidney, mainly in the tubules, which was largely suppressed by ASP (**Figure 5A**, a, b and c). ASP alone did not have an effect on the kidney (**Figure 5A**, d). Quantification of apoptosis is shown in **Figure 5B**.

Western blotting results showed that the increased expression of cleaved CASPASE-3 and cleaved PARP-1 (increased 2.5-fold and 4.1-fold compared to that in the CTRL group, respectively) in the kidneys of mice treated with LPS was significantly attenuated by ASP; the cleaved CASPASE-3 level was reduced by 47%, and the cleaved PARP-1 level was reduced by 65%. In addition, elevated kidney apoptosis in LPS-treated mice was accompanied by up-regulation of the pro-apoptotic marker BAX (increased by 5.6-fold compared to the CTRL group), accompanied by a reduction in the anti-apoptotic marker BCL-2 (decrease by 52% compared to that in the CTRL group). In the presence of ASP, the level of BAX was reduced by 63%, and BCL-2 was increased 2.9-fold (**Figures 5C, D**).

### ASP Decreased Kidney Inflammation, WNT5A, and Cell Apoptosis in LPS-Induced PE

**Figure 6:**
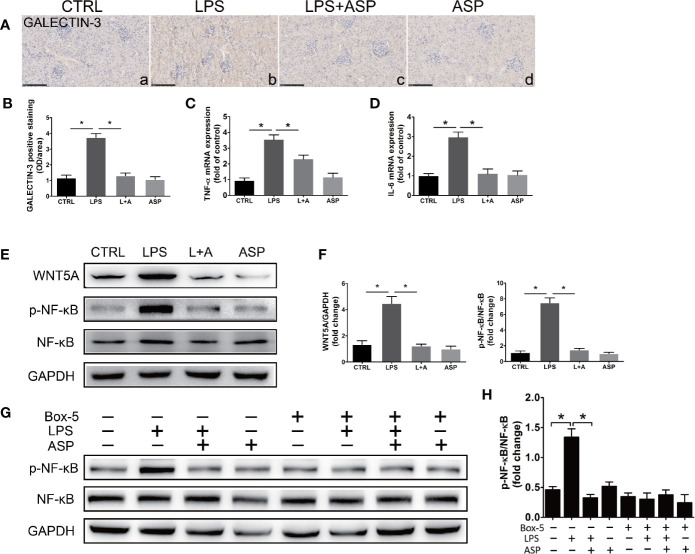
ASP ameliorated kidney inflammation in mice with LPS-induced PE. **(A)** Immunohistochemical staining for GALECTIN-3 in kidney tissues. Scale bar, 100 µm. **(B)** Histological quantification of the mean OD of GALECTIN-3 per viewing field among the four groups according to the statistical analysis. **(C, D)** Real-time PCR results showing the levels of TNF-α and IL-6 in the kidneys. **(E)** Representative western blot showing the protein levels of WNT5A, phosphorylated NF-κB, and NF-κB in the kidneys. **(F)** Bar charts showing the relative densities of the WNT5A and p-NF-κB bands. **(G)** Representative western blot results showing the levels of phosphorylated NF-κB, NF-κB in kidney primary cells upon WNT5A inhibition. The cells were obtained from pregnant mice at E18.5 in CTRL, LPS, L+A, and ASP treatment group, and six animals from each group respectively. **(H)** Bar charts showing the relative densities of the p-NF-κB bands. Data are expressed as the mean ± SEM. ^∗^Compared with the corresponding control; P < 0.05.

LPS activates the release of inflammatory cytokines in the peripheral cycle. We further investigated the effect of LPS on inflammation in the kidney. Immunohistochemistry of GALECTIN-3 marks macrophages in the kidney. As shown in **Figure 6A**, administration of ASP reduced LPS-induced accumulation of GALECTIN-3-positive cells in kidneys (**Figure 6A**, b and c). Statistical analysis showed that the mean OD of GALECTIN-3 significantly increased in the LPS-treated group compared with the CTRL group (3.5-fold). ASP reduced GALECTIN-3-positive cells in the kidneys of LPS-treated mice by approximately 64% (**Figure 6B**).

The kidney expression levels of TNF-α and IL-6 in each group were detected by qRT-PCR. LPS significantly promoted the expression of the two factors, as the TNF-α (3.4-fold) and IL-6 (2.8-fold) levels in this group were increased compared with those in the CTRL group. ASP can reduce TNF-α and IL-6 by 33% and 67%, respectively (**Figures 6C, D**). WNT5A and NF-κB are key factors in the regulation of the inflammatory response. We further examined the effect of LPS on the expression of WNT5A, activation of NF-κB in the kidney and the effects of ASP on both. The results showed that the WNT5A level and NF-κB p65 phosphorylation were significantly elevated in the LPS-treated mice compared with the CTRL mice, although no significant differences in total NF-κB were noted. Statistical analysis showed that the kidney level of WNT5A in LPS-treated mice was 2.3-fold higher than that in the CTRL mice, while the phosphorylated NF-κB p65 level in the LPS-treated group was 4.6-fold higher than that in the CTRL group. Administration of ASP rescued the up-regulation of WNT5A and the phosphorylation of NF-κB p65 in LPS-induced mice. The level of WNT5A was reduced by 59%, and the phosphorylation level of NF-κB was reduced by 71%. These results indicated that ASP attenuates kidney inflammation and inhibits NF-κB in LPS-induced PE (**Figures 6E, F**).

To further confirm the regulation of ASP is WNT5A dependent, kidney primary cells were isolated at E18.5 and treated with WNT5A antagonist Box-5 in each group, and NF-κB activation was detected. The results indicated that inhibition of WNT5A largely blocked LPS-induced phosphorylation of NF-κB. These indicated the effectiveness of ASP was WNT5A/NF-κB-dependent (**Figures 6G, H**).

### ASP Relieves HK-2 Cell Damage by Inhibiting the WNT5A/NF-κB Pathway

**Figure 7:**
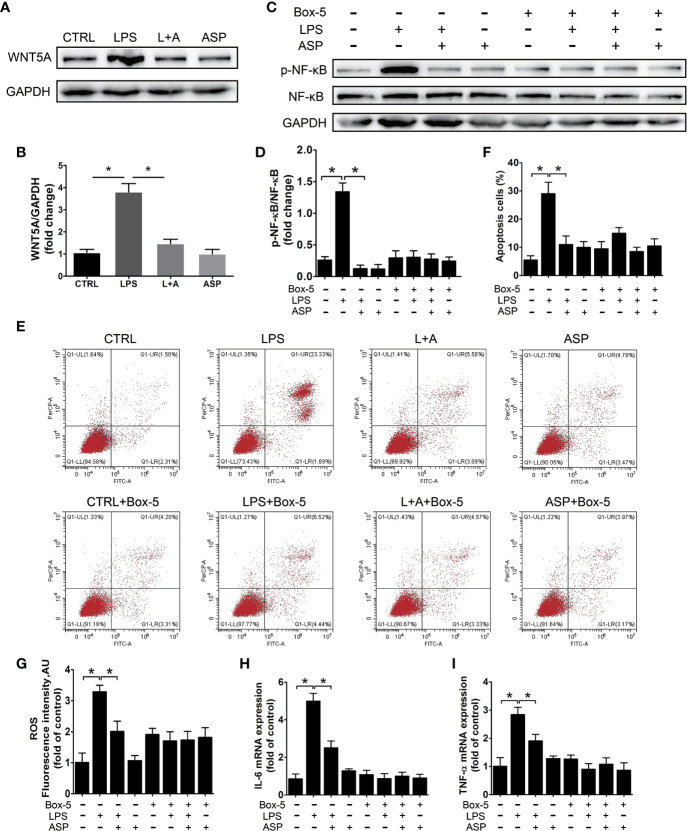
ASP relieves HK-2 cell damage dependent upon the WNT5A/NF-κB signaling pathway. **(A)** Representative western blot results showing the levels of WNT5A in HK-2 cells treated with LPS in the presence or absence of ASP. **(B)** Bar charts showing the relative densities of WNT5A bands. **(C)** Representative western blot results showing the levels of phosphorylated NF-κB, NF-κB in HK-2 cells upon WNT5A inhibition with or without LPS and ASP. **(D)** Bar charts showing the relative densities of the p-NF-κB bands. **(E)** HK-2 cell apoptosis in each group under the same treatment as in **(C)**. **(F)** The statistical results reflect the percentage of apoptosis cells in **(E)**. **(G–I)** Statistical results showing the levels of ROS, IL-6, and TNF-α in HK-2 cells under the same treatment as in **(C)**. Data are expressed as the mean ± SEM. ^∗^Compared with the corresponding control; P < 0.05.

We further evaluated the protective effect of ASP on kidney damage *in vitro*. HK-2 cells were used as an *in vitro* model to study the function of renal tubules. We analyzed activation and regulation of the inflammatory pathway by LPS and ASP, respectively. Western blot analysis showed that LPS treatment could induce the expression of WNT5A. The level of WNT5A was significantly increased by 3.5-fold with those in the CTRL group. In the presence of ASP, WNT5A expression was reduced by 55%. ASP treatment alone does not affect the expression of WNT5A (**Figures 7A, B**).

To further confirm the regulation of ASP is WNT5A dependent, HK-2 cells were treated with the WNT5A antagonist Box-5 in the presence or absence of ASP. During these procedures, NF-κB activation and cell apoptosis were detected. The results indicated that inhibition of WNT5A largely blocked LPS-induced phosphorylation of NF-κB and cell apoptosis. These results further revealed that the anti-apoptotic effect of ASP on LPS-induced HK-2 cells was WNT5A/NF-κB-dependent (**Figures 7C–F**).

Next, we examined the level of ROS and the expression of inflammatory factors in each group. Compared with the CTRL condition, LPS treatment significantly up-regulated the levels of ROS, IL-6, and TNF-α. As ROS increased by 3.2-fold, IL-6 and TNF-α were increased by 5.1- and 2.8-fold at the mRNA level, respectively. The up-regulation of ROS, TNF-α and IL-6 induced by LPS could be reduced by 38%, 45%, and 33% in the presence of ASP, respectively. However, with Box-5 co-treatment, the LPS-induced up-regulation of ROS, TNF-α, and IL-6 was largely blocked. Neither co-treatment with ASP nor ASP alone affected the levels of these factors (**Figures 7G–I**). These results indicated that ASP relieves HK-2 cells apoptosis and inflammation dependent upon the WNT5A/NF-κB signaling pathway.

## Discussion

In this study, we aimed to elucidate the mechanism involved in the intervention effect of ASP on an LPS-induced PE-like mouse model with a main focus on the mitigation of kidney damage. *In vivo* studies indicated that ASP attenuates hypertension, proteinuria, and the inflammatory response in mice with LPS-induced PE. Placental and kidney damage can be rescued by ASP. Kidney oxidative stress and renal tubule apoptosis were relieved upon ASP treatment. In addition, *in vitro* studies indicated that ASP relieves tubular epithelial damage by inhibiting the WNT5A/NF-κB pathway. These findings indicate that ASP has an anti-inflammatory effect in PE-like mouse model and rescues kidney damage by inhibiting cell inflammatory responses, apoptosis, and oxidative stress.

Studies have confirmed that ASP can prevent the occurrence of PE in high-risk women when started before 16 weeks of gestation (33). We further optimized the animal model for ASP treatment at embryonic day 9.5 (E9.5) (for the previous study, ASP treatment was at E7.5) (16). Mice placentation was initiated after implantation (E4.5) between E4.5 and E10.5 (42), and pregnant mice were treated with LPS on E7.5. Severe placental dysplasia appeared upon LPS treatment. ASP intervention began at E9.5 in mice with a dose equivalent to 100 mg/day in pregnant women with an average weight of 70 kg. Some developmental time differences exist between mouse and human placentas, although both are hemochorial. Studies shows that E3.5 in mice corresponds to approximately day 5 postimplantation in humans. Implantation occurs at E4.5 in mice, corresponding to day 7–8 postimplantation in humans. In mice, chorioallantoic attachment initiates from labyrinth formation on approximately E8.0, with branching morphogenesis is complete by E10.5, the halfway point of gestation; however, the labyrinth continues to grow throughout the latter half of gestation. In humans, villous formation starts early on approximately day 13 postimplantation, and the villi are fully vascularized by the end of the first trimester or beginning of the second trimester. At the developmental level, E10.5 in mice corresponds to week 13 in humans. Given that the mouse placenta is fully constructed at E10.5, we employed ASP at E9.5 in the mouse model, which corresponded to approximately week 13 in humans (34). During pregnancy, the weight gain in mice is much greater than that in humans, so a 15.2 mg/kg dose was administered. The PE-like phenotype was significantly rescued in the LPS+ASP group.

Acute kidney injury is one of the most prominent features of PE. However, the pathogenic mechanisms remain unclear. Abnormal placentation and endothelial dysfunction both play essential roles in PE. Placental spiral artery remodeling defects lead to abnormal perfusion of the intervillous space. Ischemia of the placenta leads to the release of antiangiogenic and vasoconstrictive factors into the maternal circulation; these factors include sFLT-1, TNF-α, and IL-6 and can induce vascular endothelial injury in distant organs, including the kidneys and other organs. Here we showed that LPS injection in pregnant mice induced TNF-α and IL-6 production, and these factors increased at E10.5 and continued to E13.5. This indicated the PE-related systemic inflammatory response lead to the kidney injury.

Albumin/creatinine represents the kidney function and is used to evaluate the glomerular filtration rate (43). Several studies confirmed the effect of ASP on PE-related proteinuria (44, 45). In this study, we found that ASP attenuated kidney dysfunction in LPS-treated mice, as shown by the reduced albumin/creatinine level and histopathological alterations. Given that LPS treatment leads to tubular injuries in the kidney, we further detected the effectiveness of ASP on the expression of KIM-1 and NGAL. The results indicated that ASP rescued the tubular injuries induced by LPS. In addition, GALECTIN-3-positive macrophages were also enhanced, indicating a high state of inflammation in the kidney. The changes were significantly rescued by ASP, implying its effectiveness in inhibiting LPS-induced inflammatory responses in the kidneys. Oxidative stress caused by pathological accumulation of ROS is involved in the development of PE-related renal injury (46). ASP mitigates oxidative stress in LPS-induced kidney damage. Apoptosis is an important cause of kidney injury, and ASP rescued LPS-induced kidney cell apoptosis. These findings strongly support the idea that ASP can be a useful drug to ameliorate LPS-induced kidney damage during pregnancy.

Abnormal activation of the inflammatory response is related to the occurrence of PE. NF-κB signaling is one main regulator responsible for controlling the release of pro-inflammatory factors and is over-activated in PE patients (47). Our previous study showed that ASP exerted inhibitory effects on LPS-induced NF-κB activation and induced the release of pro-inflammatory factors in the placenta. In this study, we investigated the role of ASP in LPS-induced kidney injury to obtain more mechanistic insight. The results showed that the WNT5A level and NF-κB phosphorylation in the kidney were significantly increased in mice with LPS-induced PE. However, these abnormal activation events were reversed by ASP.

The regulation of WNT5A in diverse inflammatory diseases is well known, but its participation in PE-related kidney damage has rarely been studied. Several studies have confirmed that WNT5A promotes cytokine expression in cancer cells (48, 49). However, few of these studies explored the mechanisms underlying WNT5A and NF-κB. Activation of NF-κB by WNT5A has been described in several cell types (50). Our data indicated that NF-κB was activated by WNT5A following LPS treatment in HK-2 cells, thus leading to cell damage. Administration of ASP markedly prevented oxidative stress, apoptosis, and inflammatory responses in cells, which was WNT5A/NF-κB-dependent. These findings offer insight into the regulation of WNT5A in PE-related kidney injury.

ASP has many physiological effects; we proved ASP prevents kidney damage in LPS-induced preeclampsia by inhibiting the WNT5A and NF-κB signaling pathways in this study. We will dive deeper in the further research to determine whether it plays a role in other pathways. In addition, reports have indicated that impaired glomerular filtration and proteinuria are also regulated by accurate crossing-talk between endothelial cells and podocytes (51, 52). Future studies will focus on renal cell function, such as nephrin shedding from podocytes and the inflammatory response in glomerular endothelial cells (53).

PE is usually associated with acute kidney injury that gradually subsides after delivery. The glomerular and endothelial dysfunction may lead to chronic kidney injury and end-stage renal disease in the future. PE therapy is basically related to the control of hypertension, proteinuria, and renal function, and the goal is to minimize maternal and fetal risks. The inflammatory response is the main cause of PE-associated kidney injury. ASP was found to exert intervention effects on LPS-induced kidney damage, mainly focusing on the inhibition of cell apoptosis and the activation of NF-κB. Given the effectiveness and safety of ASP in clinical use, further studies are needed to verify the molecular mechanism of ASP on kidney function, which will help us develop preventive, diagnostic, and therapeutic strategies for PE and related renal syndromes.

## Data Availability Statement

The raw data supporting the conclusions of this article will be made available by the authors, without undue reservation.

## Ethics Statement

The animal study was reviewed and approved by Animal Ethics Committee of Peking University Health Science Center.

## Author Contributions

GL and WW contributed equally and should be regarded as joint first authors. All authors contributed to the article and approved the submitted version.

## Funding

This work was supported by the grant from the National Key R&D program of China (2016YFC1000302), and the Beijing Natural Science Foundation (5204042).

## Conflict of Interest

The authors declare that the research was conducted in the absence of any commercial or financial relationships that could be construed as a potential conflict of interest.
