# Supplementary material for: Low-Dose Aspirin Prevents Kidney Damage in LPS-Induced Preeclampsia by Inhibiting the WNT5A and NF-κB Signaling Pathways
Source: Front Endocrinol (Lausanne). 2021 Mar 11;12:639592. doi: 10.3389/fendo.2021.639592 (PMC8006287; doi:10.3389/fendo.2021.639592)
Supplement: Supplementary file 1 [file DataSheet_1.pdf]

Figure S1

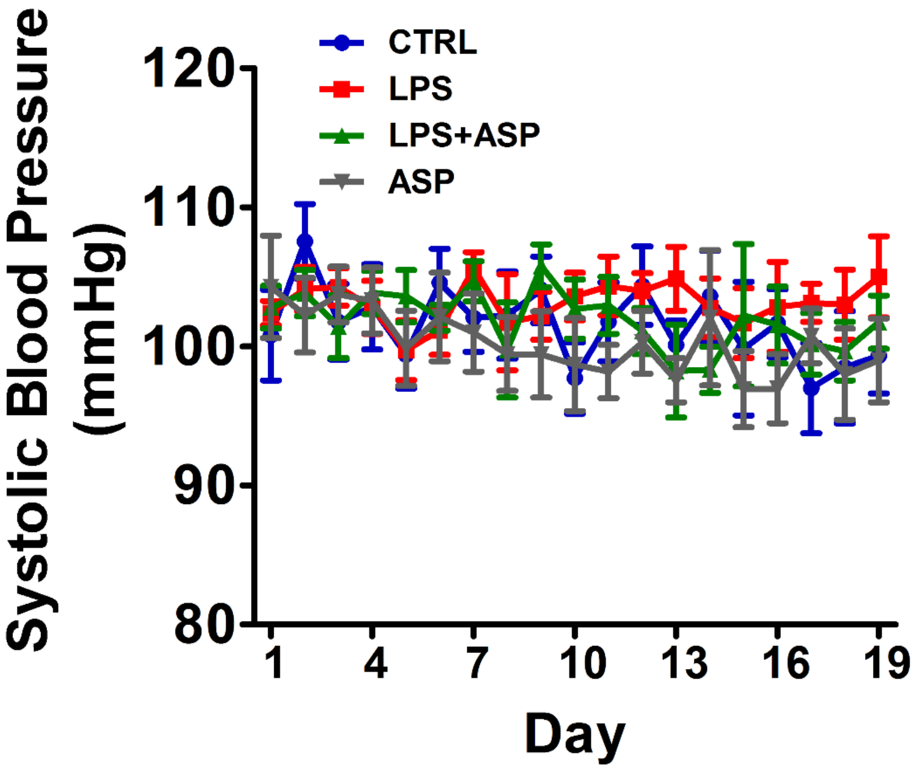

Figure S1 LPS had no effect on blood pressure in non-pregnant mice, with or without ASP treatment.

SBP was measured daily for 19 days in each group (8 animals per group). Data are expressed as the mean ± SEM.

Figure S2

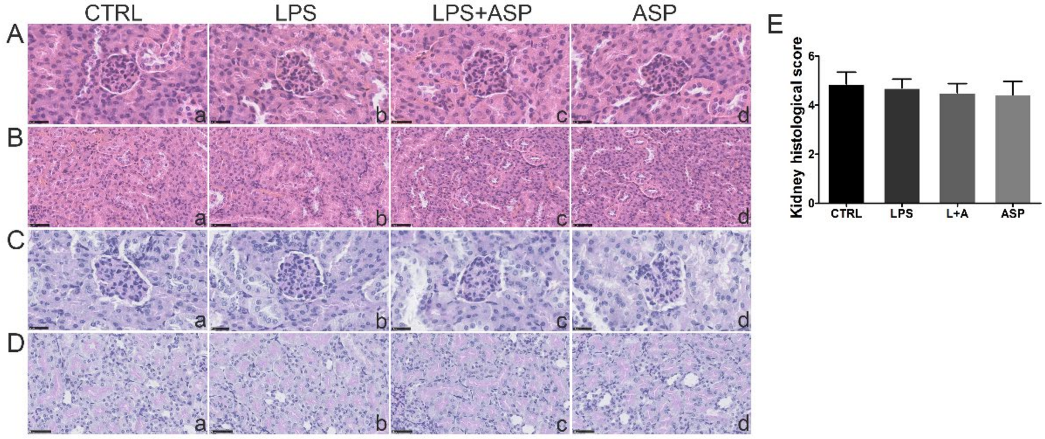

Figure S2 LPS had no effect on kidney morphology in non-pregnant mice, with or without ASP treatment.

(A and B) H&E staining indicated kidney structure in non-pregnant mice. Scale bars, 25 μm in (A) and 50 μm in (B). **(C and D)** Kidney PAS staining indicated the morphology in non-pregnant mice. Scale bars, 25 μm in (C) and 50 μm in (D). **(E)** The statistical results reflect arbitrary histological quantification of kidney histological score among the four groups (15 glomeruli for each field and 6 animals/kidneys for each group). Data are expressed as the mean ± SEM.
